# Age- and Sex-Related Differences in GFAP and UCH-L1 Levels in Mild Traumatic Brain Injury

**DOI:** 10.3390/ijms27114944

**Published:** 2026-05-29

**Authors:** Celia Espinar-Barranco, Gemma Álvarez-Corral, María del Rio-Rico, María Isabel Romero Manjón, Eva Gutiérrez, Francisco Ruiz-Cabello, Juan Francisco Gutiérrez-Bautista

**Affiliations:** 1 Programa de Doctorado en Bioquímica y Biología Molecular, University of Granada, 18016 Granada, Spain; celia.espinar.sspa@juntadeandalucia.es; 2 Servicio de Análisis Clínicos e Inmunología, Hospital Universitario Virgen de las Nieves, 18014 Granada, Spain; gemma.alvarez.sspa@juntadeandalucia.es (G.Á.-C.); mariadrr22@gmail.com (M.d.R.-R.); juanf.gutierrez.bautista.sspa@juntadeandalucia.es (J.F.G.-B.); 3 Instituto de Investigación Biosanitaria de Granada (ibs.GRANADA), 18012 Granada, Spain; 4 Servicio de Radiología, Hospital Universitario Virgen de las Nieves, 18014 Granada, Spain; mariaisabel.romero.sspa@juntadeandalucia.es; 5 Servicio de Urgencias, Hospital de Neurotraumatología Virgen de las Nieves, 18014 Granada, Spain; woselina75@gmail.com; 6 Departamento de Bioquímica, Biología Molecular e Inmunología III, University of Granada, 18016 Granada, Spain

**Keywords:** mild traumatic brain injury, GFAP, UCH-L1, blood biomarkers, computed tomography, age-adjusted cut-off values, diagnostic accuracy

## Abstract

Blood-based biomarkers such as glial fibrillary acidic protein (GFAP) and ubiquitin carboxy-terminal hydrolase L1 (UCH-L1) are increasingly used to rule out intracranial injury in patients with mild traumatic brain injury (mTBI); however, their circulating levels may be influenced by demographic factors. This study evaluated the effects of age and sex on GFAP and UCH-L1 concentrations and assessed whether adjusted cut-off values improve the diagnostic performance of head computed tomography (CT). In this retrospective observational study, 820 consecutive patients with mTBI presenting within 12 h of injury underwent CT imaging and biomarker measurements using a chemiluminescent immunoassay. CT-positive findings were identified in 11.8% of patients (n = 97). Biomarker levels were analyzed according to age and sex, and optimized cut-off values were derived and evaluated. GFAP concentrations varied significantly according to both age and sex, whereas UCH-L1 demonstrated mainly sex-related differences. The use of manufacturer-recommended fixed cut-offs resulted in markedly reduced specificity in older patients, particularly for GFAP. Age-adjusted cut-offs improved specificity while maintaining high negative predictive values across all age groups, although this was accompanied by a reduction in sensitivity and an increase in false-negative results. For the combined GFAP/UCH-L1 assay, sensitivity ranged from 90.9% to 100%, specificity from 42.6% to 57.0%, and negative predictive values from 97.7% to 100%. Age-adjusted cut-off values for GFAP ranged from 82.1 to 112.2 pg/mL across adult age groups, whereas age-adjusted UCH-L1 cut-off values ranged from 469.6 to 1072.5 pg/mL. In conclusion, age and sex significantly influence GFAP and UCH-L1 concentrations in mTBI patients. Demographic-adjusted cut-offs improve diagnostic calibration and reduce false-positive classifications while maintaining high negative predictive values despite a moderate reduction in sensitivity.

## 1. Introduction

Traumatic brain injury (TBI) represents a major public health problem worldwide, with high incidence and significant impact in terms of morbidity, disability and healthcare resource utilization [1,2]. A large majority of patients presenting to the Emergency Department after head trauma are classified as having mild traumatic brain injury (mTBI), usually defined by a Glasgow Coma Scale (GCS) score between 13 and 15. Despite the apparently mild clinical presentation, a relevant proportion of these patients may harbor intracranial traumatic lesions detectable on computed tomography (CT), some of them being clinically relevant and requiring closer monitoring or intervention [3,4,5].

TBI is not a static event but a dynamic pathological process. The initial mechanical impact produces a primary injury, characterized by immediate structural damage such as contusions, hemorrhages and diffuse axonal injury [6,7]. This primary damage triggers a cascade of secondary injury mechanisms, including neuroinflammation, blood–brain barrier disruption, cerebral edema, oxidative stress, and metabolic dysfunction, which may evolve over hours to days after the traumatic event [6,7]. These secondary processes can significantly worsen the initial injury and are often not adequately reflected by early clinical examination, particularly in patients with mTBI.

Head CT remains the reference imaging technique for the detection of acute intracranial injury after mTBI. However, the systematic use of CT in all patients with mTBI has important limitations. Most CT scans are normal, leading to overuse of imaging, increased length of stay in the Emergency Department and unnecessary exposure to ionizing radiation [1,7]. In addition, excessive use of CT contributes to Emergency Department overcrowding and increased healthcare costs [7,8].

Several clinical decision rules have been developed to reduce unnecessary CT scans and validated in both adult and pediatric populations. Although these rules generally show high sensitivity for detecting intracranial injury, their specificity is limited, and their application in real-life clinical practice is often variable [9,10]. In pediatric patients, clinical assessment can be even more challenging due to age-related symptom variability, limited communication abilities in younger children and heterogeneity in injury mechanisms [3,5].

Among the most studied biomarkers are glial fibrillary acidic protein (GFAP), a specific marker of astroglial injury, and ubiquitin carboxy-terminal hydrolase L1 (UCH-L1), a cytoplasmic neuronal protein [7,8,11]. Both biomarkers are released into the bloodstream shortly after a brain injury and can be detected within the first hours following trauma.

GFAP and UCH-L1 reflect different but complementary aspects of brain injury. GFAP is an intermediate filament protein predominantly expressed in astrocytes and plays a key role in maintaining astroglial structural integrity [12,13,14]. Astrocytic injury leads to GFAP release into the cerebrospinal fluid and subsequently into peripheral blood through disruption of the blood–brain barrier or glymphatic pathways [14]. Because astrocytic damage is closely related to secondary injury mechanisms, GFAP is considered a marker of ongoing brain tissue injury.

In contrast, UCH-L1 is a neuron-specific cytoplasmic enzyme involved in the ubiquitin–proteasome system and is essential for neuronal protein homeostasis and axonal transport [3,15]. Neuronal cell body injury results in the rapid release of UCH-L1 into the bloodstream, making it a marker of early neuronal damage [12,15].

Several observational and multicenter studies in adult populations have demonstrated that serum levels of GFAP and UCH-L1 are significantly associated with the presence of traumatic intracranial lesions on CT imaging, showing high sensitivity and high negative predictive value for ruling out intracranial injury in mTBI patients [2,9,10,11]. These findings led to the development and regulatory approval of combined GFAP and UCH-L1 in vitro diagnostic tests to support clinical decision-making in adults with mTBI [12,16]. This represented an important milestone, as these tests were the first commercially available blood-based assays specifically designed for use in mTBI.

Age has been shown to play a critical role in the interpretation of GFAP and UCH-L1 levels. Several studies have demonstrated that circulating concentrations of these biomarkers vary across a lifespan, with GFAP concentrations being especially elevated in children younger than 3.5 years and changing progressively across age groups [17,18]. In a recent cohort of adult mTBI patients, the use of manufacturer-defined fixed cut-offs (GFAP < 35 ng/L; UCH-L1 < 400 ng/L) yielded an overall sensitivity of 93.1% and a negative predictive value (NPV) of 95.5% but with limited specificity (28.8%). When age-adjusted cut-off values were applied, both sensitivity and NPV reached 100% across all age groups, with improved specificity in patients older than 50 years [17]. In pediatric populations without TBI, physiological GFAP levels show marked age dependence, with up to 20% of samples exceeding the adult diagnostic threshold and nearly all children younger than 3.5 years presenting values above the adult cut-off [18].

In addition to age, sex may represent another relevant biological factor influencing biomarker concentrations and response to brain injury. Differences in neuroinflammatory response, hormonal modulation and brain structure between males and females could potentially affect both baseline and post-traumatic levels of GFAP and UCH-L1, although this aspect has been insufficiently explored in clinical studies [19,20].

Most previous studies have evaluated these biomarkers using fixed cut-off values applied across heterogeneous populations, which may partly explain the variability observed in specificity, particularly in older patients. This study includes one of the largest single-center cohorts to evaluate age- and sex-specific cut-off values for GFAP and UCH-L1 in a real-world Emergency Department population. By integrating demographic stratification with computed tomography findings, we provide clinically relevant evidence to support a more individualized application of blood-based biomarkers in the management of mTBI.

## 2. Results

A total of 820 patients with mTBI were included. All patients met the predefined inclusion criteria and underwent head CT as part of the routine diagnostic management in the Emergency Department. The demographic characteristics and biomarker measurements of the overall study population are summarized in Table 1. Serum GFAP and UCH-L1 concentrations were available for all included patients. No missing biomarker data were observed.

**Figure 1:**
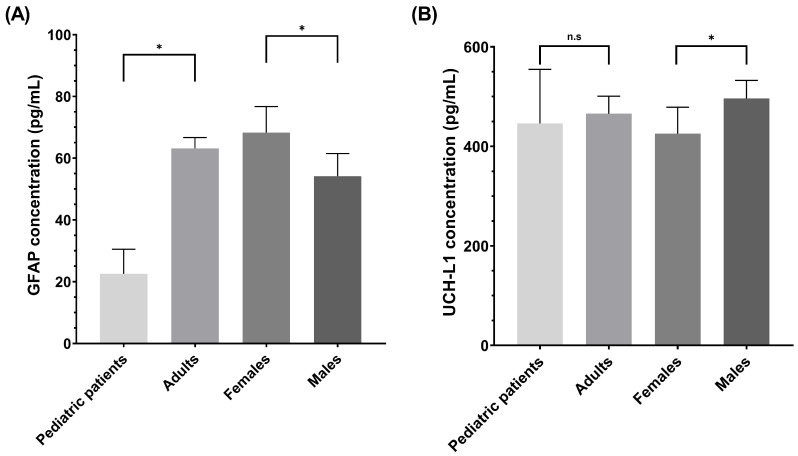
Comparisons of GFAP and UCH-L1 concentrations according to age (<18 vs. ≥18 years) and sex. (**A**) Serum glial fibrillary acidic protein (GFAP) concentrations in pediatric patients and adult patients, as well as in females and males. GFAP levels were significantly lower in pediatric patients compared with adult patients and significantly higher in females compared with males. (**B**) Serum ubiquitin C-terminal hydrolase L1 (UCH-L1) concentrations in pediatric patients and adult patients, as well as in females and males. No significant differences were observed between pediatric patients and adult patients, whereas males showed significantly higher UCH-L1 levels than females. Pediatric patients were defined as <18 years (n = 74), and adult patients as ≥18 years (n = 746). Bars represent median values, and error bars indicate interquartile range * Statistically significant (*p* < 0.05); ns, not significant. GFAP concentrations were significantly lower in pediatric patients compared with adult patients (*p* < 0.001), whereas UCH-L1 levels did not differ significantly between these two age categories. When stratified by sex, both GFAP and UCH-L1 concentrations showed significant differences between female and male patients (*p* < 0.001 and *p* = 0.01, respectively). These findings suggest that age and sex influence circulating biomarker levels and support further stratified analyses.

Given the known biological variability of brain injury biomarkers, we explored whether GFAP and UCH-L1 concentrations differed according to age and sex. The comparisons between pediatric and adult patients (<18 vs. ≥18 years), as well as between female and male patients, are presented in Figure 1.

To further explore the relationship between biomarker concentrations and imaging findings, GFAP and UCH-L1 levels were compared between CT-positive and CT-negative patients. Both biomarkers’ concentrations were significantly higher in CT-positive patients compared with CT-negative patients (median [IQR]: 98.3 (78.2–572.6) vs. 55.1 (25.4–90.9) pg/mL for GFAP; 668.5 (471.9–1862.5) vs. 424.4 (223.5–751) pg/mL for UCH-L1; *p* < 0.001 for both comparisons), indicating a clear association between biomarker elevation and the presence of intracranial injury.

Pediatric patients were excluded from subsequent diagnostic performance analyses. Data from the pediatric population are presented in Supplementary Table S1.

In light of the observed variability in biomarker concentrations, we evaluated whether age- and sex-specific optimization of cut-off values could improve diagnostic performance in relation to head CT findings. The optimized cut-off values for GFAP and UCH-L1 varied substantially across adult age groups and between female and male patients, with corresponding differences in sensitivity, specificity, and AUC, indicating that fixed thresholds may not fully capture biomarker performance in heterogeneous populations. Overall, both age and sex influenced the optimal diagnostic thresholds of GFAP and UCH-L1, supporting the need for stratified interpretation.

Adult patients were stratified into predefined age groups to account for known age-related variability in circulating GFAP and UCH-L1 concentrations and to enable a more granular assessment of diagnostic performance across the adult lifespan.

To further characterize the impact of age on biomarker performance, the diagnostic accuracy metrics for GFAP and UCH-L1 were analyzed separately across adult age groups. The age-specific optimized cut-off values and corresponding sensitivity, specificity, predictive values, and AUCs are reported in Table 2. For both biomarkers, diagnostic performance varied across age groups, although we achieved 100% sensitivity and a negative predictive value (NPV) in the 18–50-year age group. In particular, sensitivity decreased in older age groups, indicating a reduced ability to detect CT-positive cases with increasing age. However, this reduction in sensitivity was accompanied by relatively preserved or even increased specificity across all age groups. Importantly, NPV remained consistently high across all age groups. These findings suggest that although age influences sensitivity, the optimized age-specific thresholds maintain strong rule-out performance while improving overall test calibration across the adult lifespan.

When age-specific optimized cut-off values were compared with the manufacturer-recommended fixed thresholds (Supplementary Table S2), substantial differences in diagnostic performance were observed across age groups. For GFAP, the use of age-optimized thresholds markedly improved specificity, particularly in patients older than 70 years, increasing from 11.2% with the manufacturer-recommended fixed cut-off to 81.1%, while maintaining a high NPV. In younger adults (18–50 years), the optimized GFAP threshold achieved 100% sensitivity and a high NPV with improved specificity compared with the fixed cut-off approach.

For UCH-L1, age-specific thresholds also modified diagnostic performance across age strata. In patients older than 70 years, specificity increased from 40.1% to 69.4%, although with some reduction in sensitivity. Across all age groups, NPV remained consistently high for both biomarkers.

In addition, diagnostic performance was assessed separately for female and male patients. The sex-specific optimized cut-off values and corresponding performance metrics for GFAP and UCH-L1 are summarized in Table 3. Differences between sexes were observed for both biomarkers, particularly regarding specificity and overall discriminatory performance. Despite these variations, NPV remained consistently high in both female and male patients, supporting the potential clinical utility of these biomarkers while highlighting the relevance of sex-specific interpretation.

Compared with the manufacturer-recommended fixed thresholds (Supplementary Table S3), the use of sex-specific optimized cut-off values generally resulted in higher specificity and improved balance between sensitivity and specificity across sexes. This effect was particularly evident for GFAP in female patients, where specificity increased substantially from 19.0% to 82.6%, although accompanied by reduced sensitivity. Similar trends were observed for UCH-L1, with the optimized thresholds reducing false-positive classifications while maintaining a consistently high NPV. Overall, these findings suggest that sex-specific threshold adjustment may improve test calibration and reduce overclassification associated with fixed cut-off strategies. In particular, applying uniform thresholds derived predominantly from male adult populations may unnecessarily increase FP classifications in female patients.

Taken together, these findings confirm that both age and sex influence the optimal diagnostic thresholds of GFAP and UCH-L1. A comprehensive overview of age- and sex-specific optimized cut-off values and their corresponding diagnostic performance metrics is provided in the Supplementary Material (Supplementary Table S4), allowing integrated assessment of both stratification variables.

Finally, we evaluated the diagnostic performance of the combined GFAP/UCH-L1 assay using age-adjusted cut-off values across adult age groups and in the overall adult population (Table 4). The combined age-adjusted strategy maintained consistently high NPVs across all age categories, ranging from 97.7% to 100%. In the overall adult cohort, the combined assay achieved a sensitivity of 91.2%, a specificity of 59.2%, and an NPV of 98.0%, indicating a high negative predictive value with a more balanced sensitivity–specificity profile, although at the expense of reduced sensitivity, relative to the manufacturer-recommended fixed cut-offs.

Across age groups, diagnostic performance remained more stable than that observed with individual biomarkers alone, although specificity was lower in patients older than 70 years. Nevertheless, the use of age-adjusted combined thresholds reduced the imbalance associated with the manufacturer-recommended fixed cut-offs while preserving high clinical reliability in excluding intracranial injury.

Sex-stratified analysis of the combined assay using optimized cut-off values is presented in Table 5. In female patients, the combined assay achieved a sensitivity of 89.2%, a specificity of 64.2%, and an NPV of 98.0%, with an AUC of 0.77. In male patients, sensitivity reached 98.1%, the NPV was 99.3%, and specificity was 42.0%, with an AUC of 0.70. Compared with the manufacturer-recommended fixed cut-off values (Supplementary Table S3), the use of optimized sex-specific thresholds substantially improved specificity in female patients while preserving high sensitivity and NPV in both sexes. Although specificity remained moderate in male patients, the combined optimized strategy maintained robust rule-out performance and more balanced diagnostic accuracy across female and male patients.

To further evaluate the clinical impact of different cut-off strategies, we compared the numbers of false negatives (FNs), false positives (FPs), and potentially avoidable CT scans using the manufacturer-recommended thresholds, sex-adjusted cut-offs, and age-adjusted cut-offs (Table 6).

A positive result was defined as either GFAP or UCH-L1 exceeding the corresponding cut-off value. Manufacturer-recommended cut-offs correspond to GFAP ≥ 35 pg/mL and UCH-L1 ≥ 400 pg/mL. Sex-adjusted and age-adjusted cut-offs were derived from the present study. CT scans avoided were defined as the proportion of patients with negative biomarker results who did not show intracranial lesions on CT.

Table 6 summarizes the clinical impact of the different cut-off strategies on diagnostic errors and potential CT scan reduction. The manufacturer-recommended cut-off values achieved maximal sensitivity (100%) with no FN cases; however, this strategy resulted in a high number of FP cases (n = 541) and a low specificity (17.4%), allowing avoidance of only 15.3% of CT scans. In contrast, both sex-adjusted and age-adjusted strategies substantially reduced the number of FP cases and increased the proportion of potentially avoidable CT scans. Sex-adjusted cut-offs reduced the number of FP cases to 311 and increased the proportion of potentially avoidable CT scans to 46.1%, while maintaining sensitivity at 94.5%. Similarly, age-adjusted cut-offs further reduced the number of FP cases to 268 and increased the proportion of potentially avoidable CT scans to 51.9%, although sensitivity decreased to 91.2% with eight FN cases. Detailed analysis of the FN cases revealed a highly consistent clinical profile. All FN cases occurred in elderly patients older than 70 years and corresponded to small-volume intracranial hemorrhages or chronic/subacute extra-axial collections. In these cases, lesions included minimal traumatic subarachnoid hemorrhages, chronic or subacute subdural hematomas, hygromas, and small hemorrhagic foci. Only a minority of FN cases corresponded to lesions requiring neurosurgical assessment, whereas no epidural hematomas, large parenchymal contusions, or acute lesions with major mass effect were identified among FN patients. Notably, the FN cases identified using sex-adjusted thresholds largely overlapped with those observed under the age-adjusted strategy, suggesting that these missed cases were primarily driven by age-related biological and clinical characteristics rather than sex-specific effects alone.

**Figure 2:**
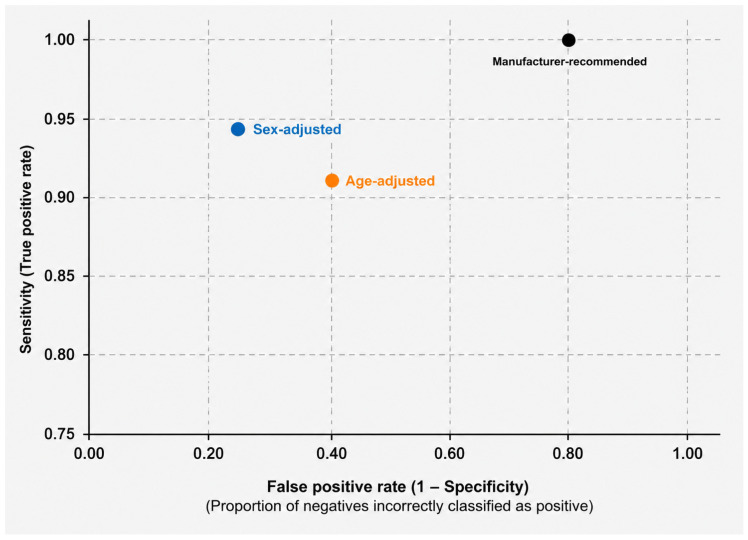
Trade-off between sensitivity and specificity for different cut-off strategies.

Overall, the optimized cut-off strategies improved specificity and substantially reduced unnecessary CT imaging, although at the cost of a small increase in false-negative classifications. This trade-off between sensitivity and specificity across strategies is visually summarized in Figure 2.

Comparison of manufacturer-recommended thresholds, sex-adjusted cut-offs, and age-adjusted cut-offs for the combined GFAP/UCH-L1 assay. Each point represents the overall diagnostic performance of a specific cut-off strategy rather than a conventional ROC curve. Age- and sex-adjusted thresholds reduce false-positive classifications at the expense of a moderate reduction in sensitivity, illustrating the balance between diagnostic safety and a reduction in unnecessary CT imaging.

## 3. Discussion

The present study demonstrates that serum GFAP and UCH-L1 concentrations in patients with mTBI vary significantly according to age and sex and that optimized diagnostic cut-off values differ substantially across demographic subgroups. These findings have important implications for the clinical application of brain injury biomarkers in Emergency Department settings and suggest that a “one-size-fits-all” approach to biomarker interpretation may not be optimal across heterogeneous patient populations.

### 3.1. Age-Related Effects on GFAP and UCH-L1 Concentrations

One of the main findings of this study is the marked influence of age on GFAP concentrations and diagnostic performance. While the GFAP concentrations in our cohort were overall lower in pediatric patients than in adult patients, previous studies have demonstrated substantial age-dependent variation within pediatric populations, with the highest GFAP levels observed during early childhood and progressive decline thereafter [17,18]. By contrast, UCH-L1 concentrations showed less pronounced age-related variation, suggesting that astroglial biomarkers may be more sensitive to developmental and age-related biological processes than neuronal injury markers [18]. The apparent discrepancy between our findings and previous pediatric studies reporting elevated physiological GFAP concentrations in young children is likely explained by differences in pediatric cohort composition. In our study, the pediatric subgroup was predominantly composed of adolescents (median age 16 years), and no children younger than 3.5 years were included. In contrast, previous studies demonstrating markedly elevated baseline GFAP levels focused primarily on early childhood populations, in whom developmental astroglial activity appears to be substantially higher [18]. Therefore, our findings should not be interpreted as contradictory but rather as reflecting different pediatric age distributions and clinical settings.

At the opposite end of the age spectrum, diagnostic specificity declined markedly in older adults, particularly for GFAP, when the manufacturer-recommended fixed cut-off values were applied. Several mechanisms may contribute to this observation, including chronic white matter disease, subclinical cerebrovascular injury, neurodegenerative processes, low-grade neuroinflammation, and age-related blood–brain barrier dysfunction [10,17,21]. These findings are consistent with previous reports demonstrating the poor specificity of fixed biomarker thresholds in elderly populations [15,17].

An important finding of this study is that the optimized age-adjusted thresholds consistently improved specificity compared with the manufacturer-recommended fixed cut-offs while preserving excellent rule-out performance in several clinically relevant subgroups. Notably, GFAP maintained 100% sensitivity and a high NPV in patients aged 18–50 years even after applying substantially higher age-adjusted thresholds, while specificity improved from 57.0% to 74.8%. Similarly, the combined GFAP/UCH-L1 assay achieved 100% sensitivity and a high NPV in patients aged 51–70 years, despite a concomitant increase in specificity compared with the fixed-threshold approaches. These findings suggest that demographic-adjusted interpretation may improve test calibration and substantially reduce false-positive classifications, although this benefit occurs at the expense of reduced sensitivity and an increase in false-negative results. Importantly, when age-adjusted cut-off values were applied to the combined GFAP/UCH-L1 assay, diagnostic performance remained robust across the entire adult population, supporting the feasibility of implementing age-adjusted thresholds at the assay level while preserving clinical usability in Emergency Department workflows. The most pronounced benefit of applying threshold adjustment was observed in elderly patients. In individuals older than 70 years, application of the manufacturer-recommended GFAP cut-off resulted in extremely poor specificity (11.2%), indicating that nearly all patients would be classified as biomarker-positive despite negative CT findings. In contrast, application of the age-adjusted thresholds for GFAP increased specificity to 81.1% while maintaining a high NPV (94.9%). Similar improvements were observed for UCH-L1 and for the combined assay, supporting the concept that the use of fixed thresholds substantially overestimates biomarker positivity in older adults.

Detailed analysis of the false-negative (FN) cases revealed a consistent clinical profile. All FN cases occurred in patients older than 70 years and predominantly corresponded to small-volume, chronic, or subacute intracranial lesions, including minimal traumatic subarachnoid hemorrhages, chronic/subacute subdural hematomas with or without minor rebleeding, hygromas, and small focal hemorrhagic lesions. Most cases remained clinically stable or improved on follow-up imaging and were managed conservatively with observation and repeat CT evaluation. However, one FN case with a chronic/subacute subdural hematoma required neurosurgical intervention after radiological progression. No epidural hematomas, large parenchymal contusions, or acute lesions with major mass effect were identified among FN patients. Overall, these findings suggest that the optimized thresholds mainly missed lower-risk lesions, although any reduction in sensitivity should be interpreted cautiously before clinical implementation.

### 3.2. Sex-Related Differences in Biomarker Performance

Sex-specific threshold adjustment also improved diagnostic calibration compared with the manufacturer-recommended fixed cut-offs, although the magnitude of benefit was less pronounced than that observed with age adjustment. The clearest effect was observed for GFAP in female patients, where specificity increased markedly from 19.0% using the fixed cut-off to 82.6% with the optimized threshold, while maintaining a high NPV (95.9%). Although sensitivity decreased compared with the manufacturer-recommended strategy, the combined GFAP/UCH-L1 assay still preserved robust rule-out performance in female patients, with a sensitivity of 89.2% and an NPV of 98.0%. These differences may reflect sex-related biological variation in astroglial activation, hormonal modulation of neuroinflammatory pathways, and biomarker release kinetics [22]. In male patients, the impact of sex-adjusted thresholds was more modest. Nevertheless, the combined assay achieved excellent sensitivity (98.1%) and NPV (99.3%) while maintaining improved specificity compared with the fixed-threshold approaches. Overall, these findings suggest that sex-specific interpretation may reduce FP classifications, particularly in female patients, although the incremental benefit appears smaller than that achieved using age-adjusted strategies.

### 3.3. Clinical Implications of Biomarker-Guided Strategies

Across all demographic subgroups, GFAP and UCH-L1 consistently demonstrated high negative predictive values, supporting their utility as rule-out biomarkers for intracranial injury in patients with mTBI [10,12]. From a clinical perspective, biomarker-guided strategies may help reduce unnecessary CT imaging, radiation exposure, Emergency Department overcrowding, and healthcare resource utilization. However, the moderate specificity and relatively low positive predictive values observed, particularly in elderly patients, indicate that positive biomarker results should not be interpreted as confirmatory evidence of intracranial injury but rather as indicators of increased risk requiring further imaging evaluation. This asymmetry in diagnostic performance does not represent a failure of these biomarkers but rather reflects their intended role as rule-out tools designed to safely exclude significant intracranial injury in low-risk patients. Integration of biomarker testing with established clinical decision rules, such as the Canadian CT Head Rule or PECARN criteria, may further enhance risk stratification [11]. The use of these biomarkers could provide additional discriminatory power in patients classified as intermediate risk by clinical criteria, potentially improving overall diagnostic accuracy and optimizing CT utilization [4].

### 3.4. Comparison with Previous Literature

Our findings extend previous studies evaluating GFAP and UCH-L1 in mTBI populations [23,24,25,26,27,28]. While prior studies consistently demonstrated high sensitivity and negative predictive values, substantial heterogeneity persists regarding optimal cut-off values and diagnostic performance. Our results reinforce recent evidence suggesting that demographic-adjusted interpretation may improve biomarker calibration, particularly in elderly patients [15,17]. While the ALERT-TBI study established thresholds of 22 pg/mL for GFAP and 327 pg/mL for UCH-L1 [12,27], our data indicate that applying fixed thresholds in a heterogeneous Emergency Department population substantially penalizes specificity, particularly in older adults. Notably, the age-adjusted GFAP thresholds identified in older adults in our cohort are comparable to those previously proposed by Ladang et al. [15], supporting the reproducibility of this observation across independent populations. Differences between our optimized thresholds and those reported in previous studies are likely related to variations in cohort composition, sample size, analytical platforms, and the broader sampling window of up to 12 h post-injury used in our study.

### 3.5. Limitations and Future Directions

Several limitations should be acknowledged. This was a single-center retrospective observational study, which may limit generalizability. Additional clinical severity indicators, such as duration of loss of consciousness or post-traumatic amnesia, were not consistently available. In addition, age stratification was based on predefined categorical groups rather than continuous age modeling. Although these strata were selected according to clinically relevant age ranges and previous biomarker literature, alternative age boundaries were not formally tested. Consequently, sensitivity analyses evaluating different age stratification approaches were not performed, and the selected thresholds should be interpreted as exploratory. CT imaging was used as the reference standard despite its limited sensitivity for subtle or diffuse traumatic lesions, particularly in the early hours after the injury. Consequently, some biomarker-positive/CT-negative patients may have harbored MRI-detectable brain injury not captured by CT imaging. In this context, elevated biomarker concentrations in CT-negative patients may not necessarily represent false-positive results but rather an underlying brain injury below the detection threshold of conventional CT. This limitation may have important implications for the interpretation of diagnostic performance metrics. In the present study, biomarker-positive/CT-negative cases were classified as false positives for statistical purposes because CT was used as the reference standard. However, some of these patients may have had true traumatic brain injury not detectable on conventional CT imaging, particularly diffuse axonal injury, microhemorrhages, or subtle traumatic abnormalities detectable only by MRI. Consequently, specificity may have been underestimated and false-positive rates overestimated, introducing potential misclassification bias inherent to CT-based diagnostic studies of mTBI biomarkers. Precise sampling times within the 12 h post-injury window were not consistently available, precluding detailed kinetic analyses. The influence of anticoagulant or antiplatelet therapy, extracranial injury, and comorbidity burden on biomarker concentrations could not be systematically assessed in this retrospective study. Finally, although elderly patients represented the subgroup with the greatest diagnostic variability, the study was not specifically powered for dedicated analyses in very elderly populations.

Finally, our study had additional methodological limitations that warrant caution. The diagnostic performance metrics reported for GFAP and UCH-L1 represent apparent performance within the derivation cohort. While bootstrapping (10,000 resamples) was applied to estimate 95% bias-corrected confidence intervals for the optimal cut-off thresholds, no internal validation or optimism-correction procedures were performed for sensitivity, specificity, or overall diagnostic accuracy estimates. Therefore, these performance metrics may be overfit to the present sample, and further external validation in larger, independent cohorts is required before clinical implementation of these thresholds is recommended.

Future research should conduct multicenter prospective validation of the demographic-adjusted thresholds developed in this study, examine integration of biomarkers with established clinical decision rules, and develop individualized probabilistic models incorporating continuous age adjustment rather than fixed categorical thresholds. Prospective characterization of discordant cases and implementation studies evaluating integration into Emergency Department workflows will be essential before routine clinical adoption. Continuous age-adjustment approaches based on probabilistic modeling rather than fixed categorical thresholds may represent a promising strategy for future biomarker-guided decision-making.

## 4. Materials and Methods

### 4.1. Study Population

This study was conducted at Virgen de las Nieves University Hospital (Granada, Spain) between March 2022 and December 2024. The study included 820 patients admitted to the Emergency Department due to an mTBI that had occurred within the previous 12 h. Seventy-four patients were children under 18 years of age, and 746 were adult patients aged 18 years or older. Patients were eligible if they presented with mild neurological symptoms, including headache, dizziness, nausea and/or vomiting, brief loss of consciousness (<30 min), amnesia, confusion, and/or disorientation.

All consecutive patients with a Glasgow Coma Scale (GCS) score between 13 and 15 who underwent head CT as part of the routine diagnostic management in the Emergency Department were considered for inclusion. The CT scans were interpreted by experienced radiologists according to standard clinical practice. A CT scan was considered positive if the radiology report identified any acute traumatic intracranial lesion, including contusions, intracranial hemorrhage, or diffuse axonal injury. Due to the retrospective design of the study, the radiologists were not systematically blinded to the biomarker results. In addition, formal interrater agreement statistics were not prospectively collected, representing a limitation of the study.

The exclusion criteria were as follows: head trauma occurring more than 12 h prior to presentation; history of any neurological or psychiatric disorder; and previous neurosurgical procedures and/or prior traumatic brain injury [10]. Prior traumatic brain injury was considered separately from other neurological disorders due to its potential long-term impact on baseline biomarker levels. In addition, patients with severe acute or chronic systemic conditions, including sepsis, pneumonia, end-stage chronic kidney disease, and other severe non-neurological disorders, were excluded from the study [21]. However, as this was a retrospective study conducted under real-world Emergency Department conditions, systematic data on anticoagulant or antiplatelet therapy use and extracranial injury severity were not uniformly available for all patients; the potential effects of these factors on circulating biomarker concentrations could not be fully assessed and should be considered a limitation of the present work.

All methods were carried out in accordance with relevant guidelines and regulations, including the Declaration of Helsinki. All study protocols were reviewed and approved by the Portal de Ética de la Investigación Biomédica, Junta de Andalucía (Approval Code: SICEIA-2025-002510). The requirement for informed consent was waived due to the retrospective nature of the study and the use of anonymized clinical data.

### 4.2. Measurements of GFAP and UCH-L1

GFAP and UCH-L1 measurements were performed in the Emergency Laboratory of the Department of Clinical Analysis and Immunology at Virgen de las Nieves University Hospital (Granada, Spain). The Emergency Laboratory provides analytical diagnostic services for both outpatients admitted to the Emergency Department and hospitalized patients.

All mTBI assays (Abbott Laboratories, Chicago, IL, USA) were performed using the immunoassay module of the fully automated integrated Alinity ci analyzer (Abbott Laboratories, Chicago, IL, USA), which is based on the chemiluminescent microparticle immunoassay (CMIA) principle. All analyses were conducted using the manufacturer’s original application and strictly following the manufacturer’s instructions. The manufacturer-recommended cut-off values for GFAP and UCH-L1 are 35.0 pg/mL and 400.0 pg/mL, respectively.

The mTBI assay was considered positive when the concentration of one or both biomarkers exceeded the corresponding cut-off value. Analytical performance was routinely monitored using dedicated internal quality controls at three concentration levels covering the clinically relevant measurement range. The manufacturer-stated limit of detection on the Alinity platform is 2.4 pg/mL for GFAP and 31.2 pg/mL for UCH-L1. Analytical assurance that the procedure was consistent with published clinical studies employing the same assay.

GFAP and UCH-L1 concentrations were measured in blood samples collected in serum separator tubes with gel (BD Vacutainer^®^, Becton Dickinson, Franklin Lakes, NJ, USA). Serum was obtained by centrifugation at 3500 rpm for 7 min at 20 °C. Sample analysis was performed within two hours after collection, and samples with a high hemolysis index were excluded from analysis.

### 4.3. Statistical Analysis

A descriptive statistical analysis was performed. Continuous variables were expressed as median and interquartile range (IQR), while categorical variables were summarized using frequency tables and percentages. The Shapiro–Wilk test was applied to assess the normality of each continuous variable.

To compare quantitative variables (GFAP and UCH-L1) between age groups (<18 vs. ≥18 years) and between sexes, the non-parametric Mann–Whitney U test was used. For comparisons across multiple age categories (≤18, 18–50, 51–70 and >70 years), the non-parametric Kruskal–Wallis test was applied. When statistically significant differences were observed, post hoc pairwise comparisons were performed using Bonferroni correction. Comparisons involving categorical variables were conducted using the chi-square test or Fisher’s exact test, as appropriate.

Optimal cut-off values for GFAP and UCH-L1 in relation to CT findings were estimated using the Liu method [29], an ROC-based optimization approach that selects the threshold maximizing the product of sensitivity and specificity. To assess the precision of these estimates, 95% bias-corrected confidence intervals were derived from 10,000 bootstrap resamples of the threshold selection procedure. This optimization was performed once within each predefined age stratum using the original dataset. Adult age groups (18–50, 51–70, and >70 years) were predefined based on clinically relevant strata commonly used in previous mTBI biomarker studies to facilitate clinically interpretable subgroup analyses across different stages of adulthood. Age was categorized rather than modeled as a continuous variable in order to simplify threshold derivation and support potential applicability in routine Emergency Department workflows. Pediatric patients (<18 years) were not included in the diagnostic performance analyses because the study was not specifically designed or powered to derive pediatric biomarker cut-off values. Consequently, all ROC analyses, cut-off optimization procedures, and diagnostic performance calculations were performed exclusively in the adult cohort (n = 746).

Bootstrapping with 10,000 resamples was performed exclusively to estimate 95% bias-corrected confidence intervals for the optimized cut-off values and diagnostic performance measures. Resampling was performed with replacement at the patient level while preserving the original data structure. Cut-off selection was not repeated within each bootstrap sample.

Therefore, the reported diagnostic performance represents apparent (internally derived) performance and was not adjusted for optimism. No internal validation procedure, such as cross-validation or bootstrap-based optimism correction, was performed.

Furthermore, diagnostic validity measures were estimated for each dichotomized biomarker using both literature-based and study-derived cut-off values, stratified by adult age groups. These measures were also calculated for the combined criterion in which either GFAP or UCH-L1 was positive, both stratified by age groups and for the overall adult population.

Because head CT was used as the reference standard, biomarker-positive/CT-negative cases were classified as false-positive results for the purpose of diagnostic performance calculations. However, CT has limited sensitivity for subtle traumatic lesions, diffuse axonal injury, and early post-traumatic abnormalities. Therefore, some biomarker-positive/CT-negative patients may have harbored MRI-detectable brain injury not identified on CT imaging. Consequently, the specificity estimates should be interpreted within the context of the known limitations of CT as the reference standard rather than as definitive evidence of an absence of brain injury.

A two-sided *p*-value < 0.05 was considered statistically significant for all analyses. Statistical calculations were performed using STATA software, version 16.1 (StataCorp, College Station, TX, USA). Diagnostic performance measures, optimized cut-off values, and AUC estimates are reported with 95% confidence intervals.

## 5. Conclusions

In conclusion, age and sex significantly influence circulating GFAP and UCH-L1 concentrations and their diagnostic performance in patients with mTBI. Demographic-adjusted cut-off values were found to substantially improve specificity and reduce false-positive classifications, particularly in elderly patients, while maintaining high negative predictive values across demographic subgroups. However, these gains were accompanied by a reduction in sensitivity and the occurrence of false-negative cases, which were exclusively observed in older patients and predominantly corresponded to small-volume or chronic intracranial lesions. Therefore, the proposed age- and sex-adjusted thresholds should be considered exploratory and should not replace existing clinical decision-making strategies without prospective external validation. Further studies are required to determine the clinical safety, operational feasibility, and real-world applicability of personalized biomarker interpretation approaches in mTBI.

## Figures and Tables

**Table 1:** Demographic characteristics, biomarker levels, and CT findings in the overall mild traumatic brain injury cohort.

Age *	73 (37)
Age groups **	
≤18 years	74 (9)
18–50 years	146 (17.8)
51–70 years	156 (19)
>70 years	444 (54.1)
Sex **	
Male	446 (54.4)
Female	374 (45.6)
GFAP (pg/mL) *	59 (76.1)
UCH-L1 (pg/mL) *	463.4 (557.1)
Positive GFAP (>35 pg/mL) **	576 (70.2)
Positive UCH-L1 (>400 pg/mL) **	470 (57.3)
Positive mTBI assay **	685 (83.5)
Positive head CT **	97 (11.8)

Note: * median (IQR) ** n (%); GFAP, glial fibrillary acidic protein; UCH-L1, ubiquitin carboxy-terminal hydrolase L1; mTBI, mild traumatic brain injury; CT, computed tomography.

**Table 2:** Age-specific diagnostic performance and optimized cut-off values of GFAP and UCH-L1 for CT findings.

**GFAP**			
Metric	18–50 years	51–70 years	>70 years
	(n = 146)	(n = 156)	(n = 444)
Cut-off (pg/mL)	82.1	93.5	112.2
	(82.1–247.7)	(93.5–143.4)	(100–212.7)
Sensitivity	100	78.6	67.3
	(100–100)	(61.5–92.9)	(55.2–79.5)
Specificity	74.8	85.9	81.1
	(67.4–82.1)	(79.7–91.4)	(77.2–84.9)
AUC	0.92	0.83	0.75
	(0.86–0.98)	(0.73–0.93)	(0.73–0.93)
NPV	100	94.8	94.9
	(100–100)	(90.7–98.3)	(92.6–97)
PPV	24.4	55.0	32.1
	(12.8–37.5)	(40–70)	(23–40.5)
**UCH–L1**			
Metric	18–50 years	51–70 years	>70 years
	(n = 146)	(n = 156)	(n = 444)
Cut-off (pg/mL)	1072.5	469.6	623.7
	(323.5–2221.3)	(362.9–496.6)	(500–965.9)
Sensitivity	72.7	75	63.5
	(42.9–100)	(58.3–90)	(50–76.9)
Specificity	81.5	60.9	69.4
	(74.3–88.1)	(52.4–69.3)	(64.8–74.1)
AUC	0.84	0.65	0.70
	(0.73–0.94)	(0.54–0.74)	(0.62–0.77)
NPV	97.3	91.8	93.5
	(93.8–100)	(85.6–97.5)	(90.5–96.2)
PPV	24.2	29.6	21.6
	(10–40)	(18.7–40.8)	(15.2–27.9)

Values are expressed as percentage or area under the curve (AUC) with 95% confidence interval (CI). GFAP, glial fibrillary acidic protein; NPV, negative predictive value; PPV, positive predictive value; UCH-L1, ubiquitin carboxy-terminal hydrolase L1.

**Table 3:** Sex-specific diagnostic performance and optimized cut-off values of GFAP and UCH-L1 for CT findings.

**GFAP**		
Metric	Females (n = 347)	Males (n = 399)
Cut-off (pg/mL)	115.5	93.5
	(100–212.7)	(74.4–153.7)
Sensitivity	70.3	75.9
	(55.2–83.8)	(63.6–86.5)
Specificity	82.6	77.4
	(78.2–86.8)	(73.1–81.7)
AUC	0.78	0.83
	(0.67–0.88)	(0.77–0.89)
NPV	95.9	95.4
	(93.3–98.2)	(92.7–97.8)
PPV	32.5	34.5
	(22.4–43.5)	(25.4–42.7)
**UCH–L1**		
Metric	Females (n = 347)	Males (n = 399)
Cut-off (pg/mL)	623.7	469.6
	(461.7–965.9)	(414.2–1072.5)
Sensitivity	67.6	75.9
	(50–81.6)	(64–86)
Specificity	72.9	51.3
	(68.1–77.9)	(46.4–56.5)
AUC	0.75	0.66
	(0.65–0.83)	(0.58–0.74)
NPV	95	93.2
	(92.1–97.5)	(89.7–96.6)
PPV	22.9	19.6
	(14.8–31.4)	(14.3–25)

Sex-specific optimized cut-off values and corresponding diagnostic performance metrics are shown. Values are expressed as percentage or area under the curve (AUC) with 95% confidence interval (CI). GFAP, glial fibrillary acidic protein; NPV, negative predictive value; PPV, positive predictive value; UCH-L1, ubiquitin carboxy-terminal hydrolase L1.

**Table 4:** Diagnostic performance of the combined GFAP/UCH-L1 assay using age-adjusted cut-off values for detection of intracranial injury in adult patients with mild traumatic brain injury.

Combined GFAP/UCH–L1 Assay				
Metrics	18–50 Years	51–70 Years	>70 Years	All Patients
	(n = 146)	(n = 156)	(n = 444)	(n = 746)
GFAP cut-off (pg/mL)	82.1	93.5	112.2	
UCH–L1 cut-off (pg/mL)	1072.5	469.6	623.7	
Sensitivity	90.9	100	92.3	91.2
	(70–100)	(100–100)	(84.4–98.2)	(84.9–96.6)
Specificity	54.1	57	42.6	59.2
	(45.9–62.3)	(48.5–65.4)	(37.7–47.6)	(55.3–62.9)
AUC	0.72	0.79	0.67	0.75
	(0.61–0.80)	(0.74–0.83)	(0.63–0.72)	(0.72–0.79)
NPV	98.6	100	97.7	98
	(95.6–100)	(100–100)	(95.2–99.4)	(96.5–99.2)
PPV	13.9	33.7	17.6	23.6
	(6.3–22–5)	(23.8–44)	(13.3–22.1)	(19.6–28.2)

Age-adjusted cut-off values were applied to the combined GFAP/UCH-L1 assay. A positive result was defined as either biomarker exceeding its respective age-specific cut-off value. Values are expressed as percentage or area under the curve (AUC) with 95% confidence interval (CI). GFAP, glial fibrillary acidic protein; NPV, negative predictive value; PPV, positive predictive value; UCH-L1, ubiquitin carboxy-terminal hydrolase L1.

**Table 5:** Sex-specific diagnostic performance of the combined GFAP/UCH-L1 assay using optimized cut-off values for CT findings in adult patients with mild traumatic brain injury.

Metrics	Females (n = 347)	Males (n = 399)
GFAP cut-off (pg/mL)	115.5	93.5
UCH–L1 cut-off (pg/mL)	623.7	469.6
Sensitivity	89.2	98.1
	(78.1–97.6)	(93.8–100)
Specificity	64.2	42
	(58.6–69.5)	(36.8–47.3)
AUC	0.77	0.70
	(0.71–0.82)	(0.67–0.73)
NPV	98	99.3
	(95.9–99.5)	(97.7–100)
PPV	22.9	20.9
	(16.2–29.9)	(16–26.1)

A positive result was defined as either biomarker exceeding its respective sex-specific cut-off value. Values are expressed as percentage or area under the curve (AUC) with 95% confidence interval (CI). GFAP, glial fibrillary acidic protein; UCH-L1, ubiquitin carboxy-terminal hydrolase L1; AUC, area under the curve; CI, confidence interval.

**Table 6:** Clinical impact of different cut-off strategies on CT utilization and diagnostic errors.

Metrics	Manufacturer-Recommended Cut-Offs	Sex-Adjusted Cut-Offs	Age-Adjusted Cut-Offs
False Negatives (FNs)	0	5	8
False Positives (FPs)	541	311	268
Sensitivity (%)	100	94.5	91.2
Specificity (%)	17.4	52.5	59.1
CT Scans Avoided (%)	15.3	46.1	51.9

## Data Availability

The data presented in this study are available from the corresponding author upon request.

## Supplementary Materials

The following supporting information can be downloaded at https://www.mdpi.com/article/10.3390/ijms27114944/s1.
