# Supplementary material for: Age- and Sex-Related Differences in GFAP and UCH-L1 Levels in Mild Traumatic Brain Injury
Source: Int J Mol Sci. 2026 May 29;27(11):4944. doi: 10.3390/ijms27114944 (PMC13256406; doi:10.3390/ijms27114944)
Supplement: Supplementary file 1 [file ijms-27-04944-s001.zip › ijms-4281584-supplementary.pdf]

**Supplementary Table S1.** Descriptive characteristics of the pediatric cohort excluded from diagnostic accuracy analyses

| Variable | Pediatric cohort (n = 74) |
|---|---|
| **Age, years** | 16.0 (14.2–17.0) |
| **Female sex, n (%)** | 27 (36.5) |
| **Male sex, n (%)** | 47 (63.5) |
| **CT–positive findings, n (%)** | 6 (8.1) |
| **GFAP (pg/mL)*** | 22.5 (12.6–51.0) |
| **UCH–L1 (pg/mL)*** | 446.1 (161.8–832.5) |

Values are presented as median (interquartile range) unless otherwise indicated.

**Supplementary Table S2.** Age-specific diagnostic performance of GFAP and UCH-L1 using manufacturer-recommended cut-off values for CT findings in adult patients with mTBI

| **GFAP** | | | |
|---|---|---|---|
| Metric | 18–50 Years (n=146) | 51–70 Years (n=156) | >70 Years (n=444) |
| Cut–off (pg/mL) | | 35 | |
| Sensitivity (95% CI) | 100 (71.5–100) | 82.1 (63.1–93.9) | 96.2 (86.8–99.5) |
| Specificity (95% CI) | 57 (48.2–65.5) | 54.7 (45.7–63.5) | 11.2 (8.3–14.8) |
| AUC (95% CI) | 0.92 (0.86–0.98) | 0.83 (0.73–0.92) | 0.75 (0.66–0.84) |
| Negative predictive value (95% CI) | 100 (95.3–100) | 93.3 (85.1–97.8) | 95.7 (85.2–99.5) |
| Positive predictive value (95% CI) | 15.9 (8.2–26.7) | 28.4 (18.9–39.5) | 12.6 (9.5–16.2) |
| **UCH–L1** | | | |
| Metric | 18–50 Years (n=146) | 51–70 Years (n=156) | >70 Years (n=444) |
| Cut–off (pg/mL) | | 400 | |
| Sensitivity (95% CI) | 81.8 (48.2–97.7) | 78.6 (59–91.7) | 84.6 (71.9–93.1) |
| Specificity (95% CI) | 57 (48.2–65.5) | 52.3 (43.3–61.2) | 40.1 (35.2–45.1) |
| AUC (95% CI) | 0.84 (0.73–0.94) | 0.65 (0.54–0.75) | 0.70 (0.61–0.78) |
| Negative predictive value (95% CI) | 97.5 (91.2–99.7) | 91.8 (83–96.9) | 95.2 (90.7–97.9) |
| Positive predictive value (95% CI) | 13.4 (6.3–24) | 26.5 (17.4–37.3) | 15.8 (11.7–20.6) |

Values are expressed as percentage or area under the curve (AUC) with 95% confidence intervals (CI). GFAP, glial fibrillary acidic protein; UCH–L1, ubiquitin carboxy–terminal hydrolase L1.

**Supplementary Table S3.** Sex-specific diagnostic performance of GFAP and UCH-L1 using manufacturer-recommended cut-off values for CT findings in adult patients with mTBI

| **GFAP** | | |
|---|---|---|
| Metric | Females (n=347) | Males (n=399) |
| Cut–off (pg/mL) | 35 | |
| Sensitivity (95% CI) | 91.9 (78.1–98.3) | 92.6 (82.1–97.9) |
| Specificity (95% CI) | 19 (14.8–23.9) | 38.3 (33.1–43.6) |
| AUC (95% CI) | 0.78 (0.68–0.87) | 0.83 (0.76–0.89) |
| Negative predictive value (95% CI) | 95.2 (86.5–99) | 97.1 (92.6–99.2) |
| Positive predictive value (95% CI) | 11.9 (8.4–16.3) | 19 (14.5–24.3) |
| **UCH–L1** | | |
| Metric | Females (n=347) | Males (n=399) |
| Cut–off (pg/mL) | 400 | |
| Sensitivity (95% CI) | 83.8 (68–93.8) | 81.5 (68.6–90.7) |
| Specificity (95% CI) | 51 (45.3–56.7) | 41.4 (36.2–46.8) |
| AUC (95% CI) | 0.75 (0.65–0.83) | 0.66 (0.58–0.73) |
| Negative predictive value (95% CI) | 96.3 (92.2–98.6) | 93.5 (88.3–96.8) |
| Positive predictive value (95% CI) | 16.9 (11.8–23.2) | 17.9 (13.3–23.3) |

Sex-specific optimized cut-off values and corresponding diagnostic performance metrics are shown. Values are expressed as percentages or area under the curve (AUC) with 95% confidence intervals (CI). GFAP, glial fibrillary acidic protein; UCH-L1, ubiquitin carboxy-terminal hydrolase L1

**Supplementary Table S4.** Age- and sex-specific optimized cut-off values and diagnostic performance of

| Gender | GFAP | 18–50 Years | 51–70 Years | >70 Years |
|---|---|---|---|---|
| Female | **n** | 41 | 51 | 255 |
| | **Cut–off** (pg/mL) | 405.9 | 125.1 | 115.5 |
| | **Sensitivity** (95% CI) | 100 (15.8–100) | 66.7 (22.3–95.7) | 69 (49.2–84.7) |
| | **Specificity** (95% CI) | 100.0 (91–100) | 88.9 (75.9–96.3) | 82.3 (76.7–87) |
| | **AUC** (95% CI) | 1 | 0.69 (0.32–0.98) | 0.77 (0.64–0.88) |
| Male | **n** | 105 | 105 | 189 |
| | **Cut–off** (pg/mL) | 82.1 | 93.5 | 112.2 |
| | **Sensitivity** (95% CI) | 100 (66.4–100) | 81.8 (59.7–94.8) | 65.2 (42.7–83.6) |
| | **Specificity** (95% CI) | 78.1 (68.5–85.9) | 86.7 (77.5–93.2) | 82.5 (75.9–88) |
| | **AUC** (95% CI) | 0.92 (0.84–0.98) | 0.88 (0.79–0.95) | 0.73 (0.59–0.87) |
| | **UCH–L1** | **18–50 Years** | **51–70 Years** | **>70 Years** |
| Female | **n** | 41 | 51 | 255 |
| | **Cut–off** (pg/mL) | 1777.0 | 471.9 | 623.7 |
| | **Sensitivity** (95% CI) | 100 (15.8–100) | 100 (54.1–100) | 65.5 (45.7–82.1) |
| | **Specificity** (95% CI) | 94.9 (82.7–99.4) | 75.6 (60.5–87.1) | 70.8 (64.4–76.6) |
| | **AUC** (95% CI) | 0.97 (0.9–1) | 0.88 (0.75– 0.98) | 0.69 (0.57–0.8) |
| Male | **n** | 105 | 105 | 189 |
| | **Cut–off** (pg/mL) | 1072.5 | 469.6 | 668.5 |
| | **Sensitivity** (95% CI) | 66.7 (29.9–92.5) | 68.2 (45.1–86.1) | 60.9 (38.5–80.3) |
| | **Specificity** (95% CI) | 80.2 (70.8–87.6) | 53 (41.7–64.1) | 71.1 (63.6–77.8) |
| | **AUC** (95% CI) | 0.81 (0.67– 0.93) | 0.55 (0.42–0.67) | 0.7 (0.58–0.82) |

GFAP and UCH-L1.

Sex- and age-specific optimized cut-off values and diagnostic performance of GFAP and UCH-L1 in adult patients with mild traumatic brain injury. Results are stratified by sex and adult age group (18–50, 51–70, and >70 years). For each subgroup, optimized cut-off values and corresponding sensitivity, specificity, and area under the receiver operating characteristic curve (AUC) are reported, using head computed tomography (CT) as the reference standard. GFAP, glial fibrillary acidic protein; UCH-L1, ubiquitin carboxy-terminal hydrolase L1; AUC, area under the curve; CI, confidence interval.
